# Family caregiver challenges in dementia care in Australia and China: a critical perspective

**DOI:** 10.1186/1471-2318-14-6

**Published:** 2014-01-23

**Authors:** Lily Dongxia Xiao, Jing Wang, Guo-Ping He, Anita De Bellis, Jenny Verbeeck, Helena Kyriazopoulos

**Affiliations:** 1 Flinders University, Adelaide, South Australia, Australia; 2 School of Nursing, Central South University, Changsha, Hunan Province, China; 3 Alzheimer’s Australia, Adelaide, South Australia, Australia

**Keywords:** Dementia, Caregivers, Dementia services, Dementia policy, Cross-cultural studies

## Abstract

**Background:**

Both Australia and China have a large proportion of people with dementia and the prevalence will triple in Australia and increase five times in China by 2050. The majority of people with dementia are reliant on family caregivers to provide daily care and to maintain the dignity in both countries. As a consequence, caregiver burden has become a major concern because of the negative impact on the care recipients’ and the caregivers’ health. It is strongly recommended that cross-national collaboration should be conducted to share experiences in fighting dementia. The aim of this study was to compare socially and culturally constructed enablers and barriers pertinent to dementia caregivers in one capital city in Australia and one capital city in China through critical reflection on the caregivers’ subjective and objective experiences for the improvement of dementia care services in both countries.

**Methods:**

Giddens’ Structuration Theory was used as a framework to guide a concurrent mixed methods design with the qualitative strand as a priority. In the qualitative strand, data were collected by focus groups and in-depth interviews while in the quantitative strand, data were collected by questionnaire survey.

**Results:**

In total 148 caregivers participated in the project with 57 of them from Australia (26 and 31 in the qualitative and quantitative strands respectively) and 91 of them from China (23 and 68 in the qualitative and quantitative strands respectively). Findings from the qualitative and quantitative strands were presented as three categories: A higher objective burden in the Chinese cohort versus a higher subjective burden in the Australian cohort; Unmet need for caregiver support in Australia and China; and Expectations for improving dementia services in Australia and for developing dementia services in China.

**Conclusions:**

Dementia policy, services and resources need to be grounded on current research evidence in an ever-changing society like China. In Australia, dementia services need to have more components of preventing or reducing caregivers’ subjective burden. As subjective burden is mediated by culture, caregiver support mechanisms should consider caregivers’ needs associated with their cultural values.

## Background

The number of people with dementia in Australia and China was 0.195 and 5.54 million in 2005 respectively. This prevalence will triple (0.664 million) in Australia and increase five times (27 million) in China by 2050 [1,2]. The majority of people with dementia are reliant on family caregivers to care for them in both countries [3]. As a consequence, caregiver burden has become a major concern because of the negative impact on the care recipients’ and the caregivers’ health [4]. However, caregiver burden can be reduced by well-designed social structures (policies and resources) in dementia care that meet the needs of people with dementia and their caregivers. Although Australia possesses comprehensive dementia services, it still strives to improve these services [5,6]. China has the largest number of people living with dementia in the world, yet has an undeveloped dementia service system [2,7]. Cross-national collaboration to share experiences in fighting dementia has been strongly recommended [3]. This article reports part of a large project that compared dementia caregivers between Australia and China with the aim of collaboratively improving dementia care.

It has been argued that caregiver burden needs to be classified into objective burden and subjective burden as factors contributing to and interventions used to modify those types of burden differ [8,9]. Objective burden is defined as time spent on care, tasks performed and financial problems faced by the caregiver, whereas subjective burden refers to the caregivers’ perceived impact of the objective burden on them [8]. These types of burden show little linear correlation and are mediated by social structures and cultural norms used to support dementia care in any country [8,9].

It is evident in the two countries that dominant values and norms in care of older people strongly influence the respective government’s political agenda, policies and resources in aged care and dementia care [5,10]. Caring for older people is seen as part of social welfare in Australia; therefore, dementia services for older people are funded and regulated by the government at all levels. Family caregivers have been recognized as part of the care workforce and entitled to support, such as dementia education programs, respite care, and a carer allowance [6]. Analysis found that these types of services and supports were associated with reduced objective caregiver burden [4,9]. However, studies in Australia also identified that one third of families who care for people with dementia did not use dementia services despite a perceived caregiver burden [11,12]. Barriers to access services and a lack of consumer-directed dementia care contributed to this situation [5,11,12].

In China caring for older people is viewed as a family’s responsibility and this is reinforced by law [10]. As the Chinese government places such importance on families providing care of older people, dementia care services are consequently undeveloped as compared to Australian services [7,13]. The traditional family role of caring for people with dementia is becoming less prominent due to rapid societal changes, such as the ‘one-child policy’, the rapid growth of internal migration for employment, and the improved social status of women in the workforce, who were traditionally home-based in previous generations [14,15]. Informal social support mainly from relatives are widely used as a main coping strategy by caregivers, but varied among households depending on the availability of the support [7,13]. Paid caregivers are often used as another coping strategy to substitute family caregivers by those who are able to afford this cost [13,16].

Studies identified behavioral and psychological symptoms of dementia (BPSD) as the number one factor contributing to objective caregiver burden [4,9]. However, not all types of BPSD will cause subjective caregiver burden, but those perceived as “challenging behaviors” that caregivers are unable to manage included agitation, aggression, hallucination and wandering [17]. Challenging behaviors can be modified through adequate interventions designed to meet the needs of people with dementia and the caregivers’ educational needs [17-19].

Research has recognized that subjective caregiver burden is multifaceted pertinent to physical, social, emotional and developmental aspects [20,21]. This type of burden is influenced by caregiver’s reappraisal of their ability to master dementia care, coping resources, and satisfaction with their caregiver role [22-24]. Other factors such as objective burden and the care recipient’s dependence level are less important in predicting subjective burden [9,20,21]. Culture has an influence on gender and a family member’s role in care of older people [25,26]. Studies identified that a caregiver of a spouse who was female experienced significantly higher subjective burden than a male who was a non-spouse [4,20,27]. However, these contributing factors to subjective burden have rarely been analyzed and compared in cross-national and cross-cultural contexts.

Culture is defined as “a learned, patterned behavioral response acquired over time that includes implicit versus explicit beliefs, attitudes, values, customs, norms, taboos, arts, and lifeways accepted by a community of individuals” [28], p. 528. Culture has a strong influence on caregivers’ motives, the use of resources or support, and coping styles in dementia care [26,29]. Australians hold individualist values that tend to encourage and emphasize individual achievements and independence [14]. In such a cultural context, caregivers have high expectations for planning and controlling dementia services to meet their desire to live an independent way of life whilst also fulfilling their caregiver duty [5,30]. Caregivers may experience subjective burden if the dementia services are unable to meet their individual needs. In addition, older Australians generally desire to live independently without their adult children in the same household [30]. Therefore, the proportion of spouse caregivers who are also older is relatively higher, and the availability of family members to share care on a daily basis is relatively lower when compared to that in China [2,3].

Chinese endorse collectivist values that rate group achievements higher than individual ones [14]. Individuals are quite actively encouraged to make sacrifices to satisfy the group goal. In addition, Chinese are influenced by Confucianism, which promotes the value of filial piety [14,31]. The core values of collectivism and Confucianism impose the duty of care of older people on family members. The proportion of adult child caregivers and the available family members to share care on a daily basis are relatively higher in China compared to those in Australia [2,3].

### Aim

The aim of our study was to compare socially and culturally constructed enablers and barriers (or rules and resources) pertinent to dementia caregivers in one capital city in Australia and one capital city in China through critical reflection on the caregivers’ subjective and objective experiences for the improvement of dementia care services in both countries. Three objectives were set out under the aim of the study: (1) comparing objective burden and subjective burden, (2) comparing the utilization of caregiver support, and (3) comparing expectations of dementia services between Chinese and Australian cohorts.

## Methods

### Theoretical framework

Giddens’ Structuration Theory was used as a framework to address the aim of the study. Structuration Theory provides one avenue for analyzing social structures that enable or inhibit dementia caregiving and for illustrating changes needed to improve services and support in ways that are realistic and practical. Social structure, as used by Giddens, refers to the ‘rules and resources’ associated with the exercise of power over people’s actions ([32], p.25). The rules in a society are either formal (legislation and policies in aged care and dementia care) or informal (cultural norms influencing caregivers’ patterned behavioral response). Resources are divided into allocative and authoritative resources, with the former concerned with the material resources (or coping resources used by caregivers such as dementia care services, formal and informal caregiver support) to enable practice, and the authoritative dealing with the capability of harnessing human activities (for example, caregiver competence). Social structures and people’s actions (or agency) are not separated as ‘a dualism’, but are viewed as ‘a duality’, inseparable and shaped by each other ([32], p.25). Structures enable the channeling of people’s actions in a specific manner, and on the other hand could also constrain people’s rational actions. As a consequence, the outcome of peoples’ actions would include both ‘intended’ and ‘unintended consequences’. Conversely, this theory acknowledges that people have the capability generated from a ‘reflexive form of knowledgeability’ to redevelop structures in order to improve practice ([32], p.3).

**Figure 1:**
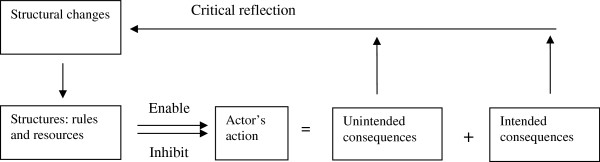
Giddens’ Structuration theory used to guide this study.

Structuration Theory is viewed as a form of critical theory used to critique structural domination and to illustrate ways to reform social structures [32]. Culture is one of the elements of a social structure and plays a key role in influencing dementia caregivers [4]. Therefore, using Giddens’ Structuration Theory as a framework in this study was relevant and showed three advantages. First, it enabled the researchers to interpret how cultural values and norms (rules) influenced caregivers’ practice in a cross-cultural context. Second, it also enabled the researchers to discuss the influence of cultural values and norms on dementia care policies and services (resources). In addition, it allowed the researchers to illuminate the direction of changes in dementia care through a collaborative form of reflection with caregivers and within a cross-national research team. Figure 1 below summarized the ‘duality’ relationship via the means of human reflexive circles based on an understanding of the theory, and was adopted from previous work in applying the theory [33].

### Research design

Structuration Theory was used as a theoretical framework to guide a concurrent mixed methods research design with the qualitative strand as a priority. The rationale of utilizing this particular research design was to capture and interpret the convergent and divergent relationships between objective and subjective caregiver burden in the cross-cultural study using multiple sources of data. Although previous studies have identified unstable relationships between these types of burden, the interpretation of the relationships is mainly grounded on quantitative findings by which the caregivers’ accounts of social-cultural conditions contributing to the relationships remain unknown. Research underpinned by critical theory has a long tradition to use both qualitative and quantitative data as triangulation to reach a comprehensive understanding of issues under study in order to facilitate changes [34].

Giddens’ Structuration Theory served as an overarching framework for data collection, integrating and interpreting findings from both the qualitative and quantitative stands. In the qualitative strand semi-structured questions were developed to elicit caregivers’ perceptions of actions and conditions affecting their practice (see Additional file 1). The qualitative strand enabled the researchers to qualitize policies, cultural norms and coping resources used by caregivers in the two countries and the caregivers’ perceived burden in a socio-cultural-political context. In the quantitative strand, objective and subjective caregiver burdens and resources used by caregivers were measured. This strand allowed the researchers to quantitize caregiver burden and social structures (policies, cultural norms and resources) used by caregivers. Findings from both the qualitative and quantitative strands were integrated to facilitate a comprehensive understanding of objective and subjective caregiver burdens in the two cultural contexts.

### Ethical considerations

The Social & Behavioral Research Ethics Committee of Flinders University approved the study for the Australian cohort (project No. 5513) and Central South University Ethics Committee approved the research for the Chinese cohort (project No. 20127801). Participants were contacted by letters that requested their voluntary participation in a focus group discussion and telephone interview in Australia and in an interview in China. Various community organizations were asked to help to distribute the letters to potential participants. The letter enclosed: a participant information sheet, a list of semi-structured questions for focus group discussion and interview and a participant’s response slip. Participants who met the selection criteria (listed in the information sheet) were asked to indicate their willingness to participate in the study by providing their contact details on the participants response slip and returning it to the researcher via a pre-paid and pre-addressed envelope. A researcher then contacted each participant by telephone to arrange a time and venue for the focus group or interview. Written informed consent was obtained prior to focus groups and interviews.

### Participants

A convenience sample of family caregiver was selected in both the qualitative and quantitative strands in the study. To be selected, the family caregiver was required to have been caring for the person with dementia and in daily contact with the person with dementia for at least one year. The caregiver could reside either in the same or a separate household. In addition, participants were able to speak fluent English in the Australian cohort and speak Mandarin in the Chinese cohort. Participants in Australian and Chinese cohorts were recruited from multiple healthcare service providers in urban areas in the two capital cities.

### Data collection

The researchers developed semi-structured questions based on a literature review and their pre-understanding of critical concepts in Structuration Theory. These questions focused on the four areas concerning dementia caregiving and targeted the aim of study (see Additional file 1): (1) Caregiver’s competencies (related to the ‘authoritative resources’ concept and targeting enablers in the aim of study); (2) Supports, resources and services caregiver received and satisfaction with these supporting mechanisms (related to the ‘allocative resources’ concept and targeted enablers in the aim of study); (3) Difficulties and challenges caregivers faced (targeted barriers in the aim of the study); and (4) Suggestions for dementia services (related to the ‘structural changes’ concept and targeted the improvement of dementia caregiving in the aim of study).

In the qualitative strand in Australia, data were collected through three focus groups comprising of 26 caregivers. Focus groups were used to suit the caregivers when they attended a caregiver support meeting. In China there were no caregiver support groups and it was difficult in getting caregivers to travel to attend focus groups. Therefore, face-to-face in-depth interviews were conducted with 23 caregivers. Data collected from focus groups were slightly different to those from face-to-face in-depth interviews as they included some of group interactions. Nevertheless, the differences did not impact on the comparisons of these two cohorts in data analyses pertinent to the aim of study. Each focus group lasted up to 120 minutes in Australia and each interview lasted up to 90 minutes in China. Same Demographic information about the caregivers and the care recipients were also collected.

In the quantitative strand, data were collected by telephone interviews with 31 caregivers in Australia and by structured face-to-face interviews with 68 caregivers in China using the “Caregiver Survey Questionnaire”. Data collected from face-to-face and telephone interviews had little difference when analyzed. The questionnaire comprises five sections: (1) Information about family carers; (2) Information about care recipients; (3) Caregiver Burden Inventory (CBI) English version [20] and Chinese versions [35]; (4) Neuropsychiatric Inventory Questionnaire (NPI-Q) English version [36] and Chinese Version [37] and (5) The usage of community care services in Australia and Social Support Rating Scale in China.

The CBI has shown adequate internal consistency reliability and appropriate content validity in both English and Chinese versions in previous studies [20,35]. The 24-item CBI was tested having a similar 5-demension of burden across the Western and Chinese cultural groups namely: (1) Time-dependence burden, (2) Developmental burden, (3) Physical burden, (4) Social burden, and (5) Emotional Burden. The only difference in factor analysis in the two cultural groups was the item described as “I’ve had problems with my marriage”. This item was correlated with items under “Social burden” in the Western cohort, but under “Emotional burden” in the Chinese cohort. Because of the small sample size, the 5-demension burden reported by Novak and Guest (1989) was used to compare the subjective burden between the two cohorts without undertaking a factorial invariance test.

The NPI-Q has been widely used to measure caregiver distress due to the BPSD in English-speaking countries and in China and shown adequate internal consistency reliability and appropriate content validity [36,37]. Caregiver distress is one of subjective burdens based on the definition used in the study. The questions about the usage of community care services in Australia were based on the availability of Australian Government funded community care services for older people and their caregivers [6]. Participants were asked to indicate whether they used these services and the frequency of use in four categories namely: (1) never use, (2) use less than 3 months in the previous year, (3) use irregularly in each month, and (3) use regularly in each month. As government-funded dementia services were undeveloped in China and caregivers mainly used informal social support as a coping resource, the Social Support Rating Scale (SSRS) was used instead. The SSRS was developed and validated in China [38]. It included four sections namely: (1) Number of social networks, (2) Family support (yes or no answer), (3) Number of family and social supports received, and (4) Help seeking (yes or no answer).

### Data analysis

Qualitative data and quantitative data were analyzed separately and then compared as a whole. In the qualitative strand, data analysis and interpretation were informed by Giddens’ four levels of understanding of how social structures enable and inhibit people’s actions ([32], p. 327). These were modified into three levels to suit the context of this study: (1) level 1: identifying social and cultural conditions enabling or inhibiting caregivers’ practice; (2) level 2: analysing unintended consequences; and (3) level 3: identifying structural changes that could improve caregiver performance in dementia care. These levels of analyses address the aim of the study by focusing on and comparing socially-culturally constructed enablers and barriers (rules and resources) pertinent to dementia caregiving and by critically reflecting on these enablers and barriers for the purpose of improving dementia caregiving. The first author and second author undertook preliminary data analysis for the Australian cohort and Chinese cohort respectively, circulated transcripts and codes to the team for crosschecking. Meetings were scheduled to discuss and reach consensus on codes, preliminary categories and final categories.

In the quantitative strand descriptive statistics were used to summarize the characteristics of caregivers and care recipients, the severity of BPSD, caregiver distress, the five dimensions of caregiver burden, the usage of dementia services, and social support. Inferential statistics were also used to compare groups in selected variables with Chi-square-test used for nominal data, Mann-Whitney U test used for non-parametric data and bivariate Pearson correlation used for correlation analysis between two interval variables in the two cohorts. Quantitative data analysis is viewed as level 1 understanding of social conditions enabling or inhibiting practice in Giddens’ Structuration Theory framework.

Findings from the qualitative and quantitative strands were integrated to address the aim of the study. A table (see Additional file 2) that reflects the three levels of understanding and the integration of qualitative and quantitative findings was created for the purposes of analysing codes from qualitative strand, summarising significant findings from the quantitative strand, and developing preliminary categories by integrating codes and quantitative findings. Two critical concepts from the theoretical framework, unintended consequences, and structural changes were used to inform the construction of final categories for the purpose of facilitating changes in dementia caregiving. The final categories highlighted two unintended consequences (categories one and two) and expectations for dementia services through structural changes (category three). The direction of changes was illuminated and discussed through the presentation and discussion of the categories.

### Trustworthiness

Criteria for trustworthiness in the qualitative and quantitative strands were made to maximize the trustworthiness. Four criteria of trustworthiness were applied to improve the qualitative strand namely: credibility, transferability and dependability, and confirmability [39]. To achieve credibility, participants were invited to review and modify their transcripts and 16 participants completed the transcript checking in Chinese cohort. In Australia findings from this study were presented to participants and gained their agreement. Codes and categories were crosschecked between the research team. Transferability addresses the applicability of the study. To fulfill transferability, the context of dementia care and the setting were explicitly discussed with participants, in the interview guide, and during data analysis using a structured framework. To achieve dependability (consistency), the study adhered to Giddens’ Structuration Theory when developing the semi-structured interview guide, coding the data, analysing the data, and presenting the categories. Confirmability in a study using critical theory refers to developing inter-subjective understanding of issues under study by participants and researchers [34]. Two levels of interpretation of issues under study guided by the Structuration Theory were undertaken to achieve confirmability: the caregivers’ explanations of social conditions affecting their practice, and the researchers’ interpretation of those conditions contributing to unintended consequences in dementia caregiving and the way to improve the conditions.

In the quantitative strand, instruments used demonstrated accepted reliability and validity in both English and Chinese versions. Research assistants who were blind to the research design were engaged to collect data and entered the data to minimize subjective bias. Data analyses and results were cross-checked by team members to minimize errors.

## Results

In total, 148 primary caregivers participated in the project with 57 of them from Australian cohort and 91 of them from the Chinese cohort. The majority of caregivers were female and aged 60 or above in the two cohorts. The Australian caregivers were older than the Chinese caregivers. The proportion of spouse caregiver in the Australian cohort was higher than that in the Chinese cohort. Chinese cohort spent a significantly longer period of time per day on care activities than their Australian counterparts. A higher proportion of the Chinese cohort experienced a financial burden as opposed to the Australian cohort. The demographic characteristics of caregivers were summarized in Table 1.

**Table 1:** Demographic information of caregivers (n = 148)

**Items**	**Focus groups/interviews n = qq49**		**Survey n = 99**		**X**^**2 **^**or Mann-Whitney U test**^**3**^
	**Aus.**^**1 **^**n = 26**	**Chi. **^**2 **^**n = 23**	**Aus. **^**1 **^**n = 31**	**Chi.**^**2 **^**n = 68**	
Male (%)	4 (15)	6 (26)	5 (16)	23 (34)	-
Female (%)	22 (85)	17 (74)	26 (84)	65 (66)	-
Spouses or partners (%)	20 (77)	13 (57)	23 (74)	31 (46)	-
Children or relatives (%)	6 (23)	10 (43)	8 (26)	37 (54)	-
Mean age (range)	69.0 (34-83)	68.0 (47-93)	68.3 (43-84)	60.5 (29-86)	P = 0.007
Mean duration in the caregiver role (range)	6.0 (2-17)	5.0 (1-16)	3.8 (1-13)	4.1 (1-14)	-
Attended dementia course (%)	22 (85)	0 (0)	27 (87)	0 (0)	-
Stay in the same house (%)	19 (73)	20 (87)	26 (84)	52 (77)	-
Hours spent on care per day^4^	-	-	14.2 (2-24)	19.5 (1-24)	P = 0.003
Perceived financial burden (%)^4^	-	-	14 (45)	53 (78)	P = 0.003
Perceived health states (SD)	-	-	2.9(0.9)	3.0 (0.7)	-
Mean numbers of family members assisting care (SD)	-	-	1.2 (1.4)	1.4 (1.3)	-

^1^Australian cohort; ^2^Chinese cohort; ^3^the tests were undertaken for survey data only; ^4^ Objective burden: Level 1 analyses in Giddens’ Structuration Theory Framework; see Additional file 2 that shows the integration of the objective burden into the findings.

The vast majority of care recipients in the Australian cohort were male while the gender of care recipients in Chinese cohort was relatively balanced. The proportion of care recipients, who were totally dependent on caregivers to provide activities of daily living, was significantly higher in the Chinese cohort than the Australian cohort. The mean number of chronic diseases was also higher in the Chinese cohort than those in the Australian cohort. The demographic information of the care recipients were summarized in Table 2.

**Table 2:** Demographic information of the care recipients (n = 148)

**Items**	**Focus groups/interviews N = 49**		**Survey N = 99**		**X**^**2 **^**or Mann-Whitney U test**^**5**^
	**Aus.**^**1 **^**n = 26**	**Chi.**^**2 **^**n = 23**	**Aus.**^**1 **^**n = 31**	**Chi.**^**2 **^**n = 68**	
Male (%)	23 (88)	11 (48)	21 (68)	34 (50)	-
Female (%)	3 (12)	12 (52)	10 (32)	34 (50)	-
Mean age of PWD^3^ (range)	79 (62-91)	78 (59-100)	78 (40-92)	77 (52-101)	-
Duration of dementia (range)	6.0 (2-17)	5.0 (1-16)	3.8 (1-13)	3.0 (1-12)	-
Total dependence for ADLs^4^ (%)	-	-	16 (52)	60 (88)	P = 0.0005
Mean number of chronic conditions (range)	-	-	1 (0-5)	2 (1-4)	P = 0.0005

^1^Australian cohort; ^2^Chinese cohort; ^3^Person with dementia; ^4^Activities of daily living; ^5^the tests were undertaken for survey data only.

Reflecting the theoretical framework, the synthesis of findings from the qualitative and quantitative stands were presented as three categories that revealed unintended consequences of caregiving as the result of unsuitable social structures in dementia care (categories one and two) and the direction of changes to improve dementia care (category three). The three categories were: (1) A higher objective burden in Chinese cohort versus a higher subjective burden in Australian cohort, 2) Unmet need for caregiver support in Australia and China, and 3) Expectations for improving dementia services in Australia and for developing dementia services in China and are explained in more detail below.

### A higher objective burden in Chinese cohort versus a higher subjective burden in Australian cohort

Managing challenging behaviors and maintaining activities of daily living were frequently mentioned in the two groups as sources of objective burden. Chinese caregivers also described financial burden pertinent to treatments and healthcare services. Australian caregivers had more resources from the public healthcare system to cope with the objective burden than their Chinese counterparts, as a caregiver stated:

He’s very much reliant on me. I can’t leave him. For 24/7 I’m with John. He’s suddenly becoming aggressive, and gets angry really with no reason at all… Then they supply me with a carer for 2 hours that is specifically for shopping on a Friday morning, and I’m finding that all these things, are giving me just that little bit of a break [AusF1P4].

Challenging behaviors described by the Chinese caregivers were more severe than that in the Australian cohort, and endangered both the caregivers and the public, as a Chinese caregiver stated in the following excerpt:

He ate food from the garbage, cursed, and hit others. We have no choice but to lock him at home. He urinated and defecated everywhere in the house: on the television, sofa, everywhere… He threw everything from the window of the second floor, everything including waste, tore the window curtains into small pieces… He did not listen to me, but bit me… It is frightful to care for him [ChiP18].

The presence of aggressive behaviors and the caregiver’s inability to manage the behaviors in China may be attributed to the lack of government funded Dementia Behavioral Management Advisory Service. This kind of services is widely used in Australia.

Chinese caregivers were also struggling to cope with the care recipients’ higher level of dependence in activities of daily living, as a caregiver stated below.

He has been bed-ridden since he suffered a hip fracture 2 years ago. I am too old to turn him on my own. He has large bedsores and I try my best to change the dressings for him. I am unable to clean him properly each time he has bowel movements; I have to wait for my daughter to come and help me. She has a job and family and is not available for most of the day [ChiP6].

This situation reflected the undeveloped community aged care services and nursing home care options for older people with a high level of dependence in China.

Most care recipients had comorbidities and needed medical treatment and hospitalization frequently in the two cohorts. The financial burden associated with medical treatments was not mentioned by the Australian cohort as Medicare or private health insurance covered any treatments. However, most Chinese caregivers were unable to afford necessary medical treatments, as one Chinese caregiver stated in the transcript following.

During that time (when he was hospitalized), he took so many medications that I could not remember the names of all the medications. [After discharge] We couldn’t afford all of the medications and decided to reduce to the essential ones to treat only his diabetes [ChiP5].

Such selected treatments contributed to complications that the care recipient developed including BPSD, hallucinations, falls and cerebrovascular accidents. The care recipient had been admitted to acute care hospitals frequently as a result of these complications.

Findings from quantitative strand showed that Chinese caregivers experienced a higher level of objective burden evidenced by significantly longer hours per day on care activities than their Australian counterparts (19.5 versus 14.2 respectively, p = 0.003, please see Table 1). Moreover, a significantly higher proportion of Chinese caregivers experienced a financial burden in comparison with their Australian counterparts (78% versus 45% respectively, p = 0.003, please see Table 1). These findings were convergent with findings from the qualitative strand.

The Australian caregivers, however, experienced a significantly higher level of subjective burden than their Chinese counterparts as evidenced by the higher scores in developmental, social, and emotional burdens (p = 0.0005, please see Table 3 and Additional file 3). Issues underlying the divergence between the objective and subjective burdens identified through the comparisons were further discussed in the discussion section.

**Table 3:** **Comparison of severity of BPSD, caregiver distress and care burden**^**1 **^**(n = 99)**

**Categories**	**Australian Med**^**3 **^**(Q1-Q3)**^**4**^	**Chinese Med**^**3 **^**(Q1-Q3)**^**4**^	**P = value**^**5**^
Total severity of BPSD^2^ (12 items)	10 (5-16)	14 (8-15)	P = 0.423
Total caregiver distress due to BPSD^2^ (12 items)	12 (5-20)	12 (7-18)	P = 0.889
Time-dependence burden (5 items)	14 (11-17)	17 (12-19)	P = 0.083
Developmental Burden (5 items)	15 (12-16)	10 (6-13)	P = 0.0005^*^
Physic Burden (4 items)	10 (8-12)	10 (5-14)	P = 0.779
Social Burden (5 items)	8 (4-12)	4 (2-6)	P = 0.0005^*^
Emotional burden (5 items)	6 (3-9)	2 (0-6)	P = 0.0005^*^

^1^Subjective burden: Level 1 analyses in Giddens’ Structuration Theory Framework; see Additional file 2 that shows the integration of the subjective burden into the findings; ^2^Behavioral and psychological symptoms of dementia (please see Additional file 4); ^3^Med = median; ^4^Q1-Q3 = interquartile range; ^5^The p-value is based on Mann-Whitney test; ^*^p <0.05.

Bivariate correlational analysis revealed factors associated with different types of burden in the two cohorts. Relating factors associated with objective burden, there was a negative correlation between time spent on care activities and the number of family members assisting in the care (r = -0.30, p = 0.017, please see Table 4) although the relationship was relatively weak. This suggested that the more the family members who shared care, the lower the level of objective burden the primary caregivers experienced. This finding was consistent with the findings from the qualitative strand that showed the amount of caregiver involvement in providing care. There was no correlation between severity of any BPSD and the time spent on care activities in both cohorts (please also see Additional file 4).

**Table 4:** Comparison of correlations between selected variables (n = 99)

**Variables**	**Australian (n = 31)**		**Chinese (n = 68)**	
	**r**	**p**	**r**	**p**
Total severity of BPSD^1^ and total caregiver distress	0.98	0.0005	0.98	0.0005*
Total severity of BPSD^1^ and time spent on care activities per day	0.74	0.690	0.65	0.600
Total severity of BPSD^1^ and time-dependence Burden	0.36	0.047*	0.37	0.002*
Time spent on care activities and physical burden	-0.04	0.800	0.40	0.003*
Numbers of family members assisting care and time spent on care activities per day	0.06	0.800	-0.30	0.017*
Numbers of family members assisting care and “Emotional Burden”	-0.13	0.450	-0.40	0.001*

^1^Behavioral and psychological symptoms of dementia; *p < 0.05.

A number of factors were correlated with subjective burdens. There was a strong positive correlation between the severity of BPSD and caregiver distress in both cohorts (r = 0.98, p = 0.0005, please see Table 3). Moreover, the severity of BPSD was also positively correlated with Time-dependence burden in both cohorts although the relationship was relatively weak (Australian r = 0.36, p = 0.047; Chinese r = 0.37, p = 0.002; please see Table 4). In the Chinese cohort there was a positive correlation between time spent on care activities and Physical burden (r = 0.40, p = 0.003, please see Table 4) and this suggested that the higher level of objective burden in the Chinese cohort may have become a trigger of subjective burden being identified. In the Chinese cohort there was a negative correlation between emotional burden and the number of family members assisting in the care (r = -0.40, p = 0.001, please see Table 4). The result indicated that the more family members who shared care, the lower the level of emotional burden the primary caregiver experienced in the Chinese social context.

### Unmet need for caregiver support in China and Australia

Unmet learning needs in dementia care were identified in both cohorts. However, the availability of educational resources varied and the two groups described different learning needs and learning patterns. All Australian caregivers had attended a 6-week standard dementia course and gained basic knowledge of dementia care, as described by a caregiver:

My GP referred us, suggesting very strongly that we contacted the Alzheimer’s Association. I did the initial course with them and have stayed and attended lots of other things here, as well as the support group [AusF1P3].

Chinese caregivers found out information on dementia by word of mouth, as stated by one caregiver:

I heard about dementia from other people. It is a kind of loss of one’s ability to understand. It is abnormal and it is not treatable… We see doctors and nurses in the Community Care Centre, but have not received any information about dementia [ChiP3].

Most caregivers identified ongoing learning needs and the need to be an educator for the rest of the family during their journey. Australian caregivers mainly used information from the Alzheimer’s Australia association to update their knowledge, as a caregiver said:

Alzheimer’s Australia was a big source of information for me. I get most of my information from them and help and support and things like that… He’s going to deteriorate 3 years after diagnoses. So there’s definitely a lot of education needed about it, absolutely without a doubt. He really struggles with 10 people talking at once and he gets confused… Well but then you’ve got to educate them [family members and friends] [AusF2P8].

Most Chinese caregivers were unable to gain information to meet their ongoing learning needs as described by a caregiver:

I want to know why she is so verbal all day and how to reduce the behavior. I could not find information I need from TV or from the Community Care Centre [ChiP1].

Most Chinese caregivers sought help from medical doctors for challenging behaviors, as a caregiver who cared for her husband with symptoms of hallucinations and verbal and physical aggression stated:

I have taken him to doctors and psychiatrists for help, but received no useful instructions. They (the doctors) only prescribed sedatives, but I decided not to give him the medication because people have told me that this type of medication could be harmful to his health [ChiP21].

The non-compliance with a treatment regime contributed to a hip fracture caused by a fall because of the hallucinations. The care recipient became bedridden and the caregiver experienced a very high burden and more so since the hip fracture. This case not only demonstrated a misunderstanding of pharmacological management of BPSD, but also revealed a low level of health literacy among Chinese caregivers. The unintended consequences in dementia treatments showed that caregivers’ education was equally important to any medical treatment.

In Australia, despite the advantages of dementia services described by participants in the qualitative strand, survey results revealed that only 19.4-41.9% of caregivers used the services on a regular basis (please see Table 5). The usage rate of a caregiver support group was also low at 38.7% of the Australian cohort. However, the usage rate of respite care was relatively high at 64.5%.

**Table 5:** **The usage of dementia services for Australian caregivers or social support for Chinese caregivers**^**1 **^**(n = 71)**

**Group**	**Dementia services or social support**	**Frequency or Mean**	**% or SD**
Australian n = 31			
	Respite care	20	64.5
	Community aged care	13	41.9
	Extended community aged care	6	19.4
	Extended community aged care-dementia	6	19.4
	Dementia education program	27	87.1
	Caregiver support group	12	38.7
Chinese n = 68			
	Support from immediate family	59	86.8
	Support from extended family	34	50.0
	No. of social network (range)	Mean 2.6 (0-4)	SD 1.4
	Utilization of available social support (range)	Mean 5.6 (2-7)	SD 1.8

^1^Usage of dementia services in Australian and informal social support in China: Level 1 analyses in Giddens’ Structuration Theory Framework; see Additional file 2 that shows the integration of the usages into the findings.

The survey among Chinese caregivers identified that caregivers mainly sought support from their immediate family (86.8%) and extended family members (50%) (Please see Table 4). Chinese caregivers who possessed multiple social networks via family members also showed a high level of utilizing informal social support (5.6 utilized among the seven available social supports). These findings were consistent with cultural norms derived from collectivism and Confucianism in the Chinese society.

### Expectations for improving dementia services in Australia and for developing dementia services in China

The two cohorts showed different expectations for dementia services reflecting cultural norms espoused in the two societies. The Australian spouse caregivers desired to maintain their independent life and social interactions with others, as one caregiver stated:

I’ve given up a lot of things I used to do, but I still insist on going to cards twice a week, I think – it’s my outing and it’s local… Keep my brain a little bit active anyway. You have to [do so] otherwise you go bananas. You’ve got to have a bit of time for yourself [AusF2P6].

Australian caregivers also had a high expectation of government funded dementia services and support, but minimal help seeking from their children:

I put him into day care 3 days a week so that he’s got contact with other people and he has to stay out of bed…I use my son occasionally and if I can’t find anybody else I’ll use my son [AusF2P9].

On the contrary, the Chinese caregivers made sacrifices for the care recipients regardless of the hardship:

He is so heavy and is dependent on me to go to toilet. It is very difficult to take him to toilet and give him shower. …I never have a good sleep at night and I am frequently waking up during the night to toilet him or change the bed if he is wet [ChiP5].

Chinese caregivers also had a high expectation of their children to share the care, as a caregiver stated:

I am not doing these [care activities] as I am too old… My daughter and sons wash her if she is wet. She has faecal and urinary incontinence. My daughter has lived with us in order to care for her. My son also comes to help every day. His house is nearby [ChiP4].

The most mentioned expectations for government funded dementia care by Chinese caregivers included affordable treatment for dementia, rehabilitation services, respite care, and community aged care as in the following:

I hope dementia treatment can be covered by the medical insurance… I wish that nurses from the Community Care Centre would offer training programmes on dementia care [ChiP16].

I wish that the Community Care Centre would provide a day-care service for people with dementia, just like the child care centre in the community. This would allow me to leave the house to do the things I have to do [ChiP20].

In Australia, difficulties in navigating dementia and aged care services were perceived as barriers to accessing services and caregivers expected equity and access in dementia care as below.

Now I was offered a package late last year from Western Carers which is connected with the Commonwealth Carers… and yet talking to my friends, some of them know nothing about how to access them. There should be a communication channel for all carers to know available services [AusF1P6].

Australian caregivers were also unsatisfied with service provider-directed services and they preferred consumer-controlled services, as an Australian caregivers described below:

What was worrying me [was] when we went so many times backwards and forwards to get someone from XXX’s [a service provider] to join us at these meetings in time to discuss my husband’s behavioral problems…We should have more control of when and how to use the services based on our needs [AusF3P2].

Caregivers also suggested the key component of a caregiver support that worked for them as described in the following:

We need a professional facilitator like Mary (carer support coordinator from Alzheimer Association) in order to get the most from the discussion [AusF2P6].

I’ve joined Carers South Australia and that’s not just for dementia. …I don’t use it actually [AusF2P7].

The component of the caregiver support group has implications for the improvement of caregiver support as discussed in the discussion section. The major findings were summarized in three categories. First, Chinese caregivers experienced a higher level of objective burden than their Australian counterparts. This finding reflected the decline of family support and underdeveloped dementia services in China. Australian caregivers, however, experienced a higher level of subjective burden than the Chinese cohort. The findings indicated a higher expectation for meeting caregivers’ individual needs in dementia care in Australia. Second, BPSD contributed to two categories of subjective burden in both groups: caregiver distress and time-dependence burden. Third, caregivers from both groups showed an underutilization of dementia services or informal social supports whilst at the same time experiencing caregiver burden. The Australian cohort expressed expectations for improving dementia services while the Chinese cohort strongly suggested the development of basic dementia services.

## Discussion

A number of factors might have contributed to the higher level of objective burden the Chinese cohort in this study experienced. First, people with complex health issues live at home and are cared for by family caregivers as aged care facilities are not available or accessible [13,31]. In this study in the Chinese cohort the care recipients showed a higher level of dependence and more chronic diseases than those in the Australian cohort. Therefore, the Chinese cohort in this study needed to perform more in number and more complex care tasks, which were evident in the qualitative findings. Second, dementia services used to relieve caregiver burden that are available in Australia have not been established in China. The undeveloped dementia services may ultimately contribute to the higher level of objective burden among the Chinese cohort. Third, this study also identified that objective burden contributed to subjective burden in the Chinese cohort evidenced by the positive correlation between time spent on care activities and Physical Burden. The finding refutes previous studies in the Western countries stating that objective burden is less important in the prediction of subjective burden [9,24].

By comparing the two cultural cohorts in a cross-national context, this study captured the divergence between the objective and subjective caregiver burdens: a higher level of subjective burden with a lower level of objective burden in the Australian cohort versus a lower level of subjective burden with a higher level of objective burden in the Chinese cohort. The qualitative findings revealed that the Australian cohort showed a higher expectation for independence, individual achievements and socialization in an individualistic culture. The higher level of subjective burden mirrored the perception of a failure to fulfill their cultural way of life. Therefore, this study supports the influence of individualism in the care of older people in the literature [24,26,29]. The lack of tailored dementia services to meet individual needs may also have contributed to the situation. The underutilization of dementia services widely reported in the literature was mainly attributed to the lack of information to access services and the inflexible services [5,11,12]. This study revealed that the lack of design to target caregiver’s subjective burdens might also have contributed to the underutilization of dementia services. In addition, the Australian cohort showed a relatively higher proportion of female caregivers and spousal caregivers than those in the Chinese cohort. As discussed before, gender and spousal status are predictors of subjective burden. These factors might have contributed to the result in this study. Due to small sample size, this study was unable to ascertain these factors, suggesting this for future studies.

The lower level of subjective burden in the Chinese cohort may reflect these caregivers’ acceptance of cultural norms influenced by collectivism and filial piety, thereby they were more tolerant of subjective burdens [25]. The findings that the more family members who shared care, the lower the level of emotional burden the primary caregiver experienced in the Chinese cohort, suggested that the collectivistic culture may be a counter factor of subjective burdens. However, this protector may not be seen in China in the near future because of the decline in the availability of family caregivers.

In this study BPSD contributed to two types of subjective burden in both cohorts namely caregiver distress and Time-Dependence Burden. The estimated prevalence rate of BPSD in the community setting was 61–88% in Australia where people with dementia were often admitted to residential care facilities [17]. The prevalence of BPSD is estimated to be higher in China due to the undeveloped aged care facilities and the lack of Dementia Behavioral Management and Services. Numerous studies have reported that challenging behaviors can be reduced via caregiver education programs and Dementia Behavioral Management and Services or counselling services [4,17]. The untreated aggressive behaviors and their harmful consequences for the care recipients, caregivers and the public in the Chinese cohort are evidence to support the policy makers and the public health authorities to set up dementia as a priority public health agenda [3]. Although the Chinese cohort reported more severe BPSD in the qualitative strand, there was no statistically significant difference of the severity of BPSD measured by the NPI-Q between the two cohorts in the quantitative strand. Small sample size and sampling bias may mainly contribute to the result.

In China the number of older people living in an empty nest had increased by more than 9% during the past 10 years, reaching 31.77% of the population in 2010 [40]. This suggests that informal social support via kinship is no longer a viable option for caring for people with dementia at home and developing dementia services through the public healthcare system is imperative. Services used to relieve the objective burden in Australia need to be considered by Chinese authorities.

### Limitations of the study

This study has several limitations. First, the sample size in the quantitative strand was small and convenience samples living in urban areas in two capital cities. Therefore, findings from the quantitative strand can be associated with sampling bias. Second, both Australia and China are countries with multicultural populations. Many cultural groups in the two countries did not belong to either an individualist or collectivist culture. Therefore, findings cannot be generalised in other cultural groups. Third, this study used a mixed methods design underpinned by a critical theory. Therefore, findings cannot be generalised, but may be transferrable to similar social and cultural contexts in the two countries under study. Fourth, the Australian cohort showed a relatively higher proportion of female caregivers, spousal caregivers and was older than those in the Chinese cohort. These demographic factors might have confounded analyses of the study, suggesting that future studies to ascertain those factors in dementia caregiving were needed. In addition, the use of the Structuration Theory to interpret structural domination in family caregiving restricted the researchers in analysing issues outside of this theory. Therefore, the findings of the present study represent only one of many critical perspectives on socially and culturally constructed dementia caregiver challenges and the direction for improving dementia care both in China and Australia.

## Conclusions

The critical paradigm underpinned by Structuration Theory enabled this study to identify structural constraints on caregiving for persons with dementia in Australia and China by comparing, interpreting and integrating findings from both qualitative and quantitative data from two cohorts. The theoretical framework also allowed identification of directions of structural changes in dementia care and services by analyzing caregivers’ needs, perceptions and expectations for dementia services and support in both countries. Due to small sample size in the quantitative strand and sampling bias, findings cannot be generalized and further studies are needed to examine the quantitative findings in a wider population in the two countries.

Findings have implications for policy makers and service providers when planning or developing dementia care. First, dementia policy, services and resources should be grounded on current research evidence. In an ever-changing society like China, unintended consequences of dementia caregiving is inevitable if presuming that family caregivers are able to care for older people with dementia without developing dementia services and resources to support them in the public healthcare system. Second, in countries with well-developed dementia services such as Australia, the services need to be advanced to have more components of preventing or reducing caregivers’ subjective burdens. Third, as subjective burden is mediated by culture, caregiver support mechanisms should consider caregivers’ needs associated with their cultural values.

### Consent

Written informed consent was obtained from the patient for the publication of this report and any accompanying images.

## Abbreviations

BPSD: Behavioral and psychological symptoms of dementia; CBI: Caregiver burden inventory; NPI-Q: Neuropsychiatric inventory questionnaire; SSRS: Social support rating scale.

## Competing interests

The authors declare that they have no competing interests.

## Authors’ contributions

LX and GPH designed the study. JW, JV, LX and ADB carried out most of the data collection. LX undertook most of the data analysis and wrote the first draft of this paper. All authors contributed to data analysis, interpretation, critically commented on and approved the final version of the manuscript.

## Pre-publication history

The pre-publication history for this paper can be accessed here:

http://www.biomedcentral.com/1471-2318/14/6/prepub

## Supplementary Material

Additional file 1 Semi-structured questions for interviews. Click here for file

Additional file 2 Data Analysis based on Giddens’ Structuration Theory (Selected). Click here for file

Additional file 3 Comparison of caregiver burden (n = 99). Click here for file

Additional file 4 Comparison of severity of behavior and caregiver distress (n = 99). Click here for file
