# Supplementary material for: Family caregiver challenges in dementia care in Australia and China: a critical perspective
Source: BMC Geriatr. 2014 Jan 23;14:6. doi: 10.1186/1471-2318-14-6 (PMC3904419; doi:10.1186/1471-2318-14-6)
Supplement: Additional file 1 — Semi-structured questions for interviews. [file 1471-2318-14-6-S1.doc]

## Additional file 1 - Semi-structured questions for interviews

| No. | Questions |
| --- | --- |
| 1 | What do you know about dementia? |
| 2 | How do you feel about caring for a person with dementia? |
| 3 | Can you tell us about the person with dementia you care for – their history? |
| 4 | Do you know any care services for a person with dementia and for you as a caregiver? If yes, please tell us about these services and whether you are satisfied with these services? |
| 5 | Could you please make your suggestions of how to improve these services if you are not satisfied with them? |
| 6 | As a caregiver for the person with dementia, have you received any support? If yes, could you please tell us what sort of support you usually receive? |
| 7 | Have you learned how to care for a person with dementia? If yes, could you please tell us where you learned and if you found the programs were helpful for you? |
| 8 | What sort of difficulties or challenges do you face when caring for a person with dementia at home? |
| 9 | Are you a solo caregiver for the person with dementia you care for, or do you share care with other family members? How do you share the care with other family members? |
| 10 | What sort of medical treatment the person with dementia is being taken? What other diseases the person with dementia has? Do you usually seek medical treatment for the person with dementia you care for? |
| 11 | Do you have any suggestions for health professionals, health service providers or policy makers in order to better support the person with dementia to stay at home? If yes, could you please tell us your suggestions? |
| Giddens’ critical concepts framed three concerning areas in the interview questions:   1. Caregiver’s competencies (knowledge, skills and attitudes) in dementia care (related to ‘authoritative resources’ concept and enablers in the aim of the study): Questions 1,2, 3, 10 2. Supports, resources and services caregiver received and satisfaction with these supporting mechanisms (related to ‘allocative resources’ concepts and enablers in the aim of the study): Question 4, 6, 7, 9 3. Difficulties and challenges caregivers faced (related to barriers in the aim of the study) 4. Suggestions for dementia services (related to ‘structural changes’ concept and improvement of dementia caregiving in the aim of the study): Question 5, 11 | |
