# Supplementary material for: Family caregiver challenges in dementia care in Australia and China: a critical perspective
Source: BMC Geriatr. 2014 Jan 23;14:6. doi: 10.1186/1471-2318-14-6 (PMC3904419; doi:10.1186/1471-2318-14-6)
Supplement: Additional file 2 — Data Analysis based on Giddens’ Structuration Theory (Selected). [file 1471-2318-14-6-S2.doc]

## Additional file 2 - Data Analysis based on Giddens’ Structuration Theory (Selected)

| Examples of transcripts from qualitative strand | Level 1: Identifying conditions affecting caregivers’ practice (related to rules and resources enabling or inhabiting practice) | | | Level 2: Unintended consequences | | Level 3: Structural changes | |
| --- | --- | --- | --- | --- | --- | --- | --- |
|  | Codes from qualitative strand | Findings from quantitative strand (see table 1, 3, 5) | Preliminary Categories: integrated findings from the two strands | | Final categories | | |
| He ate food from the garbage, cursed, and hit others. We have no choice but to lock him at home. He urinated and defecated everywhere in the house: on the television, sofa, everywhere… [ChiP18].  With 24/7 I’m with John. He’s suddenly becoming aggressive, and gets angry really with no reason at all… Then they supply me with a carer for 2 hours that is specifically for shopping on a Friday morning, and I’m finding that all these things, are giving me just that little bit of a break [AusF1P4]. | Severe behaviour problems; Lock the person with dementia at home  Behaviour problems; Emotional reaction; Received respite | The Chinese Cohort showed a higher level of objective burden than the Australian cohort.The Australian cohort showed a higher level of subjective burden than the Chinese cohort. | Endanger the person with dementia and others;  Feeling ashamed  Lack of dementia services to relieve objective burden for the Chinese cohort  Lack of supporting mechanism to relieve subjective burden for the Australian cohort | | Higher objective burden in China but higher subjective burden in Australia | |  |
| I have taken him to doctors and psychiatrists for help, but received no useful instructions. They (the doctors) only prescribed sedatives, but I decided not to give him the medication because people have told me that this type of medication could be harmful to his health [ChiP21].  He’s going to deteriorate 3 years after diagnoses. So it definitely there’s a lot of education needed about it, absolutely without a doubt. He really struggles with 10 people talking at once and he gets confused… Well but then you’ve got educate them [family members and friends] [AusF2P8]. | Prescribe antipsychotic medications; A low level of health literacy; No component of education for caregivers  Unable to cope with deterioration; need to be prepared as an educator for family members and the public | Underuse of dementia services in the Australian cohort.  Underuse of informal social support in the Chinese cohort. | Technical approach to behavioral management by health professionals  Need for ongoing education and coaching  Need for tailored caregiver support | | Unmet need for caregiver support in China and Australia | |  |
| I hope dementia treatment can be covered by the medical insurance… I wish that nurses from the Community Care Centre would offer training programmes on dementia care [ChiP16].  Now I was offered a package late last year from Western Carers which is connected with the Commonwealth Carers…and yet talking to my friends, some of them know nothing about how to access them. There should be a communication channel for all carers to know available services [AusF1P6]. | Demands for financial support and dementia education  Demands for information supply and equal access |  | Lack basic dementia services  Dissatisfied with current services | |  | | Expectations for improving dementia services in Australia and for establishing dementia services in China |
