# Supplementary material for: Family caregiver challenges in dementia care in Australia and China: a critical perspective
Source: BMC Geriatr. 2014 Jan 23;14:6. doi: 10.1186/1471-2318-14-6 (PMC3904419; doi:10.1186/1471-2318-14-6)
Supplement: Additional file 3 — Comparison of caregiver burden (n = 99). [file 1471-2318-14-6-S3.doc]

## Additional file 3 - Comparison of caregiver burden (n=99)

| Items | Statements | Australian | | Chinese | |
| --- | --- | --- | --- | --- | --- |
|  |  | Mean | SD | Mean | SD |
|  | ***Time dependence burden*** | 13.6 | 4.3 | 15.0 | 4.6 |
| 1 | My care-receiver needs my help to perform many daily tasks. | 2.9 | 1.2 | 3.1 | 1.2 |
| 2 | My care-receiver is dependent on me. | 3.4 | 0.8 | 3.4 | 0.9 |
| 3 | I have to watch my care-receiver constantly. | 2.5 | 1.2 | 3.3 | 1.2 |
| 4 | I have to help my care-receiver with many basic functions. | 2.8 | 1.0 | 2.9 | 1.3 |
| 5 | I don’t have a minute’s break from my caregiving chores. | 2.0 | 1.4 | 2.3 | 1.3 |
|  | ***Developmental burden*** | 13.8 | 3.6 | 9.9 | 5.0 |
| 1 | I feel that I am missing out on life. | 2.6 | 0.9 | 1.9 | 1.3 |
| 2 | I wish I could escape from this situation. | 2.6 | 1.0 | 1.7 | 1.3 |
| 3 | My social life has suffered. | 2.6 | 1.1 | 1.9 | 1.4 |
| 4 | I feel emotionally drained due to caring for my care-receiver. | 2.9 | 1.0 | 2.2 | 1.3 |
| 5 | I expected that things would be different at this point in my life. | 3.1 | 1.0 | 2.2 | 1.2 |
|  | ***Physical burden*** | 10.3 | 3.1 | 9.5 | 4.9 |
| 1 | I’m not getting enough sleep. | 3.1 | 1.2 | 2.4 | 1.5 |
| 2 | My health has suffered. | 2.1 | 1.1 | 2.4 | 1.4 |
| 3 | Caregiving has made me physically sick. | 2.1 | 1.1 | 1.9 | 1.4 |
| 4 | I’m physically tired. | 3.1 | 1.0 | 2.8 | 1.3 |
|  | ***Social burden*** | 8.2 | 4.2 | 4.5 | 3.6 |
| 1 | I don’t get along with other family members as well as I used to. | 1.4 | 1.1 | 0.9 | 1.2 |
| 2 | My caregiving efforts aren’t appreciated by others in my family. | 1.6 | 1.2 | 0.5 | 1.0 |
| 3 | I’ve had problems with my marriage. | 1.3 | 1.2 | 0.2 | 0.7 |
| 4 | I don’t do as good a job at work as I used to. | 2.5 | 1.3 | 2.2 | 1.5 |
| 5 | I feel resentful of other relatives who could but do not help. | 1.5 | 1.2 | 0.7 | 1.1 |
|  | ***Emotional burden*** | 6.7 | 4.1 | 3.6 | 4.5 |
| 1 | I feel embarrassed over my care-receiver’s behaviour. | 1.5 | 1.2 | 0.6 | 1.2 |
| 2 | I feel ashamed of my care-receiver. | 0.8 | 0.9 | 0.3 | 0.9 |
| 3 | I resent my care-receiver. | 1.1 | 1.0 | 0.6 | 1.1 |
| 4 | I feel uncomfortable when I have friends over. | 1.3 | 1.1 | 0.6 | 1.0 |
| 5 | I feel angry about my interactions with my care-receiver. | 2.0 | 1.2 | 1.5 | 1.5 |

Note: scoring 0= strongly disagree, 1=disagree, 2=neither agree or disagree, 3=agree, 4= strongly agree
