# Supplementary material for: Family caregiver challenges in dementia care in Australia and China: a critical perspective
Source: BMC Geriatr. 2014 Jan 23;14:6. doi: 10.1186/1471-2318-14-6 (PMC3904419; doi:10.1186/1471-2318-14-6)
Supplement: Additional file 4 — Comparison of severity of behavior and caregiver distress (n = 99). [file 1471-2318-14-6-S4.doc]

## Additional file 4 - Comparison of severity of behavior and caregiver distress (n=99)

| Items | Behavioral and psychological symptoms | Incidence rate | | Severity | | Caregiver distress | |
| --- | --- | --- | --- | --- | --- | --- | --- |
|  | of dementia | Frequency (%) | | Mean (SD) | | Mean (SD) | |
|  |  | Australian | Chinese | Australian | Chinese | Australian | Chinese |
| 1 | Delusions | 5 (16.1) | 31(45.6) | 1.4(0.5) | 2.1(0.7) | 2.0(1.0) | 2.5(1.2) |
| 2 | Hallucinations | 7(22.6) | 33(48.5) | 1.6(0.5) | 2.1(0.6) | 2.1(1.6) | 2.5(1.2) |
| 3 | Agitation or Aggression | 20(64.5) | 29(42.6) | 1.8(0.9) | 1.6(0.7) | 2.7(1.6) | 1.7(1.0) |
| 4 | Depression or Dysphoria | 19(61.3) | 44(64.7) | 1.6(0.7) | 2.0(0.4) | 2.3(1.0) | 2.4(1.0) |
| 5 | Anxiety | 17(54.8) | 42(61.8) | 2.1(0.9) | 1.9(0.7) | 2.9(1.0) | 2.2(1.1) |
| 6 | Elation or Euphoria | 7(22.6) | 27(39.7 | 2.1(0.7) | 1.7(0.5) | 1.9(1.5) | 1.3(0.8) |
| 7 | Apathy or Indifference | 25(80.6) | 47(69.1) | 2.2(0.7) | 2.1(0.5) | 2.4(1.2) | 2.5(0.9) |
| 8 | Disinhibition | 14(45.2) | 19(27.9) | 2.1(0.7) | 2.0(0.7) | 2.1(1.3) | 2.4(1.3) |
| 9 | Irritability or Lability | 18(58.1) | 32(47.1) | 1.8(0.9) | 1.0(0.5) | 2.1(1.5) | 2.3(1.2) |
| 10 | Motor disturbance | 13(41.9) | 33(48.5) | 1.8)0.9) | 2.1(0.6) | 1.7(1.5) | 2.3(1.1) |
| 11 | Nighttime behaviors | 22(71.0)) | 36(52.9) | 1.6(0.8) | 2.1(0.6) | 1.8(1.7) | 2.6(1.2) |
| 12 | Appetite and Eating | 22(71.0) | 34(50.0) | 1.5(0.7) | 1.9(0.6) | 1.9(1.6) | 2.1(1.4) |
